# Protein–protein association properties of human βB2‐crystallins

**DOI:** 10.1002/prot.26547

**Published:** 2023-07-17

**Authors:** José‐Luis Velasco‐Bolom, Laura Domínguez

**Affiliations:** ^1^ Universidad Nacional Autonoma de Mexico, Facultad de Quimica Ciudad de Mexico Mexico

**Keywords:** βB2‐crystallins, cataract, coarse‐grained, deamidation, Markov state model, molecular dynamics

## Abstract

Protein–protein association events are involved in many physiological and pathological processes. Cataract disease is a pathology that manifests protein aggregation of crystallins. β‐Crystallins are present in a high proportion in the eye lens. Therefore, the structural study of the dimerization properties of crystallins can shed light on the first stages of protein aggregation. In the present work, we examine the protein–protein association profiles of the human βB2‐crystallin by employing extensive coarse‐grained molecular dynamics (CG‐MD) and the Markov state analysis. Interestingly, our results clearly show important changes in the protein dimerization kinetics between wt‐HβB2C and the deamidated systems. The two systems show dimeric conformations. However, the association and dissociation rates are very different. Our results show that the deamidated system can associate faster and dissociate slower than the wt‐ HβB2C. The deamidated system is in a slightly opened conformation with the Greek‐key motifs well folded, suggesting that a complete unfolding of the protein is not required for aggregation. Our results describe the first stages of crystallin aggregation due to post‐translational modifications.

## 1 INTRODUCTION

The crystallins modulate the refractive index and maintain the transparency of the eye lens.^1^, ^2^ This function correlates with their correct fold in a solution environment.^3^ However, modifications in the tertiary structure of crystallins, due to mutations and post‐translational modifications, can originate insoluble aggregates that are observed in cataract disease.^4^ Cataract is one of the most common causes of blindness and generally is considered an aging pathology.^5^, ^6^

**FIGURE 1:**
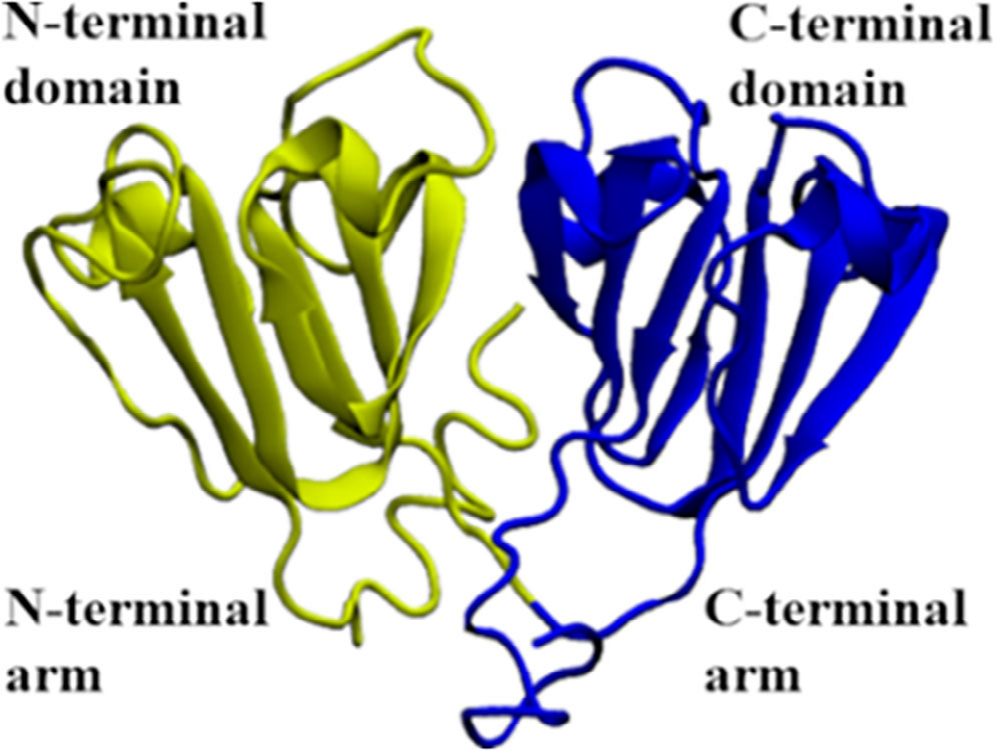
3D representation of the bilobed structure of the Human βB2‐crystallin. In green, we represent the N‐terminal domain, and in blue, the C‐terminal domain.

Crystallins are divided into two major families, α and βγ‐crystallins.^7^ α‐Crystallins are small heat‐shock proteins that form oligomeric complexes and act as a molecular chaperone for the βγ‐crystallins.^8^, ^9^, ^10^ The βγ‐crystallin family shares a common bilobed structure composed of four Greek key motifs intercalated within each domain. However, the β‐crystallins also have longer N‐ and C‐terminal extensions, which could have a role in the protein dimerization^11^(Figure 1). β‐Crystallins can associate with dimers, tetramers, and higher‐order complexes.^12^ On the other hand, γ‐crystallin are monomeric proteins and does not have long terminal extensions.^13^

βB2‐crystallin is the most abundant protein within the β‐crystallin family in the human eye.^14^ βB2 is composed of N‐terminal (N‐td) and C‐terminal (C‐td) domains connected by a short intra‐domain linker and possesses both N‐ and C‐terminal extensions (arms). βB2 can form homodimers, heterodimers, and other oligomers.^12^ A dimmer with swapped‐domain conformation has been observed in the crystallographic structure of the human βB2‐crystallin (HβB2C).^15^ However, recent experimental and computational works reported that the most stable structure of the monomeric HβB2C in a solution environment is a closed conformation, similar to other members of the βγ‐crystallin family.^16^, ^17^

On the other hand, post‐translational modifications such as deamidations have been reported as agents for developing cataracts.^18^, ^19^ The effects of glutamine deamidation have also been studied in HβB2C, observing a decrease in protein stability.^20^ However, molecular details of crystallin dimerization remain elusive.

In this work, we evaluated the protein–protein associations of wt‐HβB2C and the double deamidated HβB2C (Q70E/Q162E) employing extensive coarse‐grained molecular dynamic simulations and Markov state models (MSM). Interestingly, our results clearly show that both systems can form homodimers. However, the associated and dissociated macrostates occur at different transition rates. Furthermore, the equilibrium between the associated and dissociated conformations was changed in the Q70E/Q162E system, favoring the dimmer formation. Conversely, the wt‐HβB2C system exhibits an equilibrium between the associated and dissociated states.

## 2 METHODS

### 2.1 Initial structures and model preparation

Considering previous works,^16^, ^17^, ^21^ both wt‐HβB2C and mutated systems were built in a closed conformation using Modeller v9.22^22^ software, using a monomer of the X‐ray structure of human β‐B1 crystallin (PDB ID: 1OKI, at 1.4 Å of resolution) as a template.

### 2.2 Coarse‐grained molecular dynamics

A coarse‐grained (CG) system for HβB2C and Q70E/Q162E was generated using the martinize2.py program with the Martini v3.0 force field.^23^ Each system was solvated with Martini water beads and 150 mM NaCl. We applied an elastic dynamic network with a force constant of 700 kJ mol^−1^ nm^−2^ to the β‐sheets of the proteins and removed any force for the loop regions. The systems were minimized using the steepest‐descent algorithm and subsequently equilibrated for 20 ns under the NVT and NPT ensembles. Finally, we performed fifteen 5 μs MD simulations for each built system, yielding to 75 μs of total MD production. The temperature was set to 310 K using V‐rescale,^24^ and the pressure was set to 1.0 bar using the isotropic Parrinello–Rahman barostat algorithm.^25^ Electrostatics was accounted for using a shift function with a Coulomb cut‐off of 1.1 nm. The Lennard‐Jones potential was calculated within a cut‐off radius of 1.1 nm, with an integration time step of 20 fs. All simulations were performed using the GROMACS 5.1.4 package.^26^

### 2.3 Building Markov state models and their validation

MSMs were built with the PyEMMA v2.5.6 software^27^ to identify the kinetically relevant metastable states and their inter‐conversion rate from all trajectories. This analysis involves a featurization of the trajectories followed by the time‐lagged independent component analysis (TICA)^28^ in order to reduce dimensionality. In the next step, all the conformations from MD simulations were clustered into microstates with the k‐means algorithm. In the next step, the microstates were grouped into metastable states. The resulting discretized trajectories were used to construct the MSM. Additionally, a Chapman–Kolmogorov test (C–K test) was used to validate the constructed model^29^. The C–K test measures the predicted residence probability of each macrostate obtained from MSM with those directly computed from MD simulations. Furthermore, the free energies for each metastable state (*S*_*i*_) were computed from its stationary MSM probability *π* using the relation:

$$\mathrm{\Delta G}\left(S_{i}\right)=-k_{B}T\ln\left(\sum_{j\in S_{i}}\pi_{j}\right)$$

where *π*_*j*_ denotes the MSM stationary weight of the jth macrostate, *k*_*B*_ is the Boltzmann constant, and *T* is the temperature. Finally, the transition path theory was used to analyze the statics of the transition pathways between the macrostates. The mean first passage time (MFPT) between each pair of macrostates was calculated using the MFPT calculation module of the PyEMMA v2.5.6 software.^27^

### 2.4 Back‐conversion and all‐atom molecular dynamics

The representative structures of the macrostates obtained after MSM were subjected to back‐conversion from CG to all‐atom using the *backward.py* script.^30^ Subsequently, we selected three late dimers macrostates of the wt‐HβB2C and Q70E/Q162E, respectively, and each dimmer was solvated in a cubic box using a three‐point water model (TIP3P), and 150 mM of NaCl was added. Each structure was protonated at neutral pH and subjected to energy minimization using the steepest‐descent algorithm to remove any steric clashes. Following this, each system was equilibrated for 2 ns under NVT and NPT ensembles. The molecular dynamics production was 50 ns. The temperature was set to 310 K using the Nose‐Hoover^31^, ^32^ thermostat algorithm and the pressure was set to 1.0 bar and coupled isotropically using the Parrinello–Rahman^25^ barostat algorithm. Periodic boundary conditions in all directions were utilized to reduce finite system size effects. The Lennard‐Jones potential was calculated within a cut‐off radius of 1.0 nm. Long‐range electrostatic interactions were computed using the Particle Mesh Ewald (PME) method.^33^ An integration time step of 2 fs was used along with the LINCS^34^ algorithm to constraint covalent bonds involving hydrogen atoms. All simulations were performed with the GROMACS 2020^26^ package and the all‐atom AMBER99SB‐ILDN^35^ protein force field.

## 3 RESULTS AND DISCUSSION

### 3.1 HβB2C shows reversible protein dimerization

Most biological functions involve protein–protein interactions. Depending on the protein concentration, the β‐crystallins can associate into oligomeric complexes in vivo.^36^, ^37^ Experiments of size‐exclusion chromatography of the lens extracts showed three classes of β‐crystallin complexes based on size: βH (octamers of 160–200 kDa), βL1 (tetramers of 70–100 kDa), and βL2 (dimers of 46–50 kDa).^12^, ^38^ The β‐crystallins have longer N‐ and C‐terminal extensions, which could have a role in protein–protein interactions with other β‐crystallins.^11^ These dimeric conformations may play a critical role in eye lens transparency by maintaining the solubility between homo‐ and hetero β‐crystallin dimers.^39^ However, experimental data showed that βA3‐ and βB2‐crystallins appear to associate reversibly into homo‐ or hetero‐dimers, which might then either dissociate into monomers or further associate into multimeric complexes.^37^ The protein–protein association, their binding stability, and kinetics can be described in a free energy landscape. The MSM effectively identifies the kinetically relevant states and the interconversion rates between them.^40^ Recent studies have also reported the successful use of MSM to describe the relevant macrostates of protein–protein associations.^41^, ^42^, ^43^, ^44^

To study the protein dimerization of HβB2C, we performed extensive coarse‐grained molecular dynamics (CG‐MD) simulations of the wt‐HβB2C. The starting configuration was in the dissociated confirmation when each monomer was separated ~8.0 nm. Next, we simulated 15 replicas of 5000 ns yielding 75 μs of total CG‐MD production. Following this, all trajectories were analyzed using PyEMMA v2.5.6 software.^27^ The feature to characterize the protein–protein associations was the distance between the center of mass of each monomer domain (N‐ and C‐terminal). Subsequently, we performed a time‐lagged independent component analysis (TICA) on the feature selected to reduce the dimensionality of the feature space, using a lag‐time of 5.0 ns and preserving 95% of the kinetic variance. Next, all conformations from our CG‐MD simulations were clustered into 250 microstates using the k‐means algorithm. These resulting discretized trajectories were used to construct the MSM. Thus, the microstates were grouped into 6 metastable states using the Perron‐cluster cluster analysis (PCCA++) method. To validate our constructed model, we performed a C–K test (Figures S4 and S5). Finally, the free energy surface of the dimmer formation was computed and projected into the first two TICA components.

**FIGURE 2:**
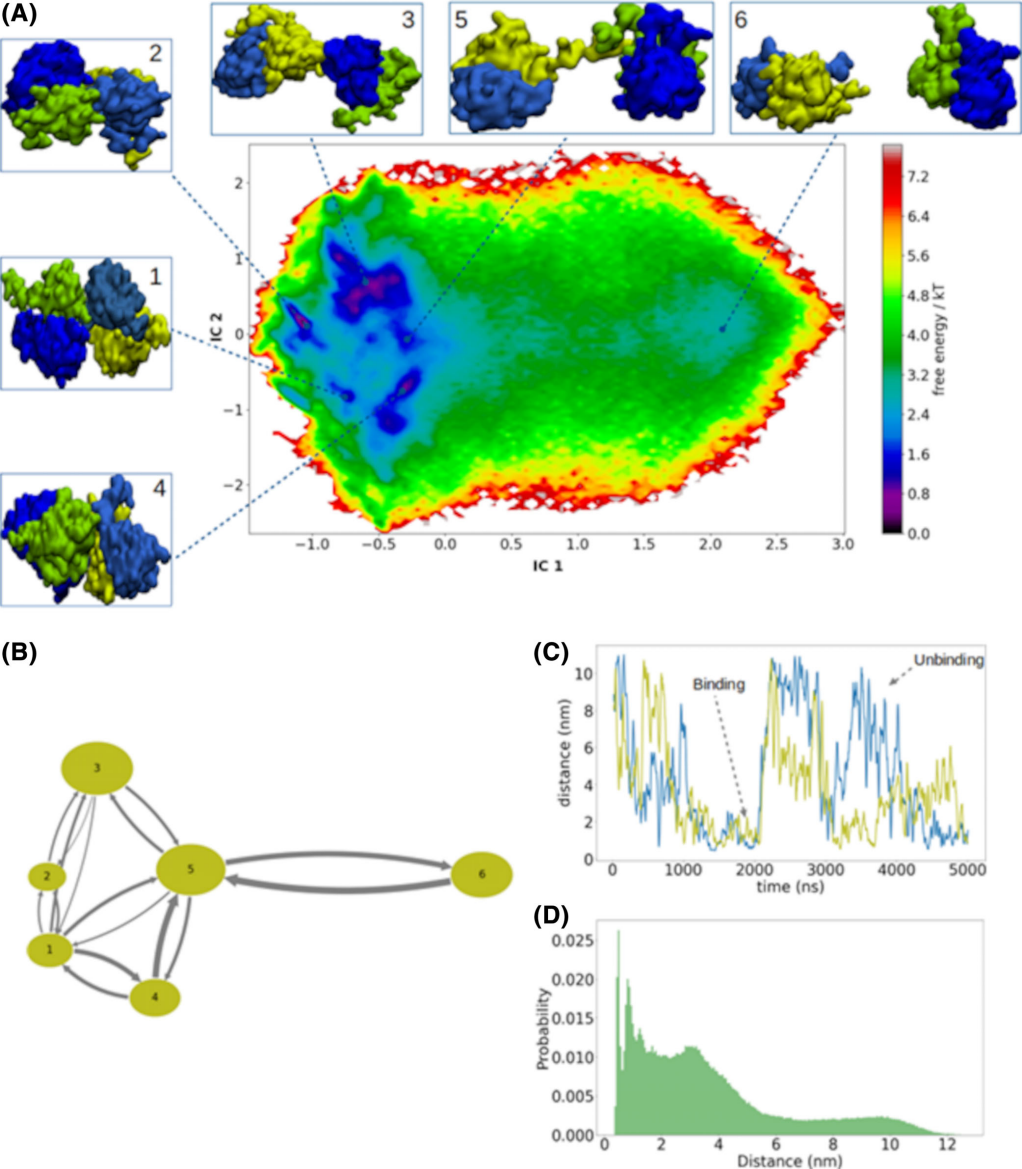
(A) Free energy surface and metastable states of the wt‐HβB2C, computed from the stationary MSM probability and projected onto the first two independent components (IC); also shown the representative structure of the relevant macrostates found in the of wt‐HβB2C dimerization process; The structures were colored, as follow: in green and yellow‐green represents the N‐terminal domain of each monomer, in blue and sky‐blue represents the C‐terminal domain of each monomer respectively. (B) Transition rates from each macrostate; the size of the circles represents the number of conformations in the respective macrostate, and the width of the arrows indicates the transitions between states, with their thickness proportional to the transition probability. (C) Two representative replicas indicating binding and unbinding events. (D) Fraction of conformations of all replicas at different distances.

Our MSM analysis reveals the interesting macrostates of the wt‐HβB2C dimerization process. It is noteworthy that the coarse‐grain force field smooths the free energy potential and only found six relevant macrostates (Figure 2A). We identify one metastable state in a dissociated conformation (macrostate 6), two metastable states as early intermediates (macrostates 3 and 5), and three states in late dimers conformations (macrostates 1, 2, and 4). Interestingly, in the macrostate 5, the N‐terminal arms allow the protein–protein association. Experimental data suggested that the N‐ and C‐ terminal arms can be essential in the HβB2C dimerization.^11^ Our results suggest that N‐ and C‐terminal arms facilitate the contact between each monomer to promote their dimerization. Macrostate 3 is also interesting because the interaction is through the N‐terminal domain (Ntd) of one monomer and another monomer's C‐terminal domain (Ctd), respectively. In macrostate 2, one monomer interacts with the cleft of the hydrophobic interface of another monomer. A conversion from CG to all‐atom resolution, shows that these interactions are stabilized mainly by hydrogen bond and salt bridge interactions as depicted in Figure S1. In macrostate 4, the dimer interaction is through the hydrophobic interface between two monomers in an antiparallel position. Macrostate 1 shows a conformation when the dimer is established by the contact between the corner of the hydrophobic interface of each monomer (Ntd of one monomer and Ctd of another monomer). This interaction is established mainly due to the side chain hydrogen bonds and salt bridges, as shown in Figures S2 and S8A.

As described above, the dimerization of the wt‐HβB2C exhibits interesting and diverse structures. However, to evaluate the association (binding) and dissociation (unbinding) events and the transition rates, we applied the transition path theory and calculated the mean first passage time (MFPT) between each pair of macrostates. The initial association event is facilitated by the N‐ and C‐ terminal arms within the first microsecond (Figure 2A–C). The transition from macrostate 6 to 5 (Figure 2B), is important for the passage from monomeric to early dimeric state. The computation of mean first passage time (MFPT) in the direction of macrostate 6 to 5, yield 418.5 ns, and in the direction of macrostate 5 to 6 is 520.7 ns. Indicating that the formation of the early dimeric stage and their dissociation can occur with a difference of 100 ns, and both states may coexist with approximately the same lifetimes. This observation is consistent with many experimental works that report that the monomeric state of wt‐HβB2C is also present.^37^, ^45^ Following the formation of the early dimeric stage, the transition to other dimeric conformations is favored (Figure 2B). The transitions between macrostates (1, 2, 3, and 4) were also observed (Figure 2B). Macrostates 1, 2, and 4 were assigned as late dimers, and also exhibit transitions between them. However, macrostate 4 is the preferable stage. From this stage can transition favorably to macrostate 5 and then it can be dissociated. An estimation of the distance between each monomer of two representative replicas displays that the binding and unbinding events can remain stable for hundreds of nanoseconds (Figure 2C). This observation is consistent with our MFT calculation. On the other hand, a high population in dimeric conformations (distance <6 nm) and a considerable population in the monomeric conformations (distance >7 nm) were observed (Figure 2D).

A back‐conversion from CG to all‐atom was performed on all macrostates Figures S1, S2, and S3 with the *backward.py* script.^30^ To evaluate the stability of late dimers, the macrostates 1, 2, and 4 were selected for all‐atom MD simulations. The computation of Root‐Mean Square Deviation (RMSD) shows that the dimers are stable over 50 ns of MD simulations (Figure S6A). Following this, the protein–protein contact maps were calculated. The results show that macrostate 1 is stabilized by at least six interactions, including ARG88‐ASP126 and ARG88‐ASP127 (Figure S8A). The macrostate 2 is stabilized by at least seven interactions, including GLU69‐ARG159 and ARG88‐GLU77 (Figure S8B). The macrostate 4 have at least four interactions, including GLU77‐LY100, and PHE26‐TRP194 (Figure S8C).

### 3.2 Deamidated HβB2C association kinetics favor dimeric states

**FIGURE 3:**
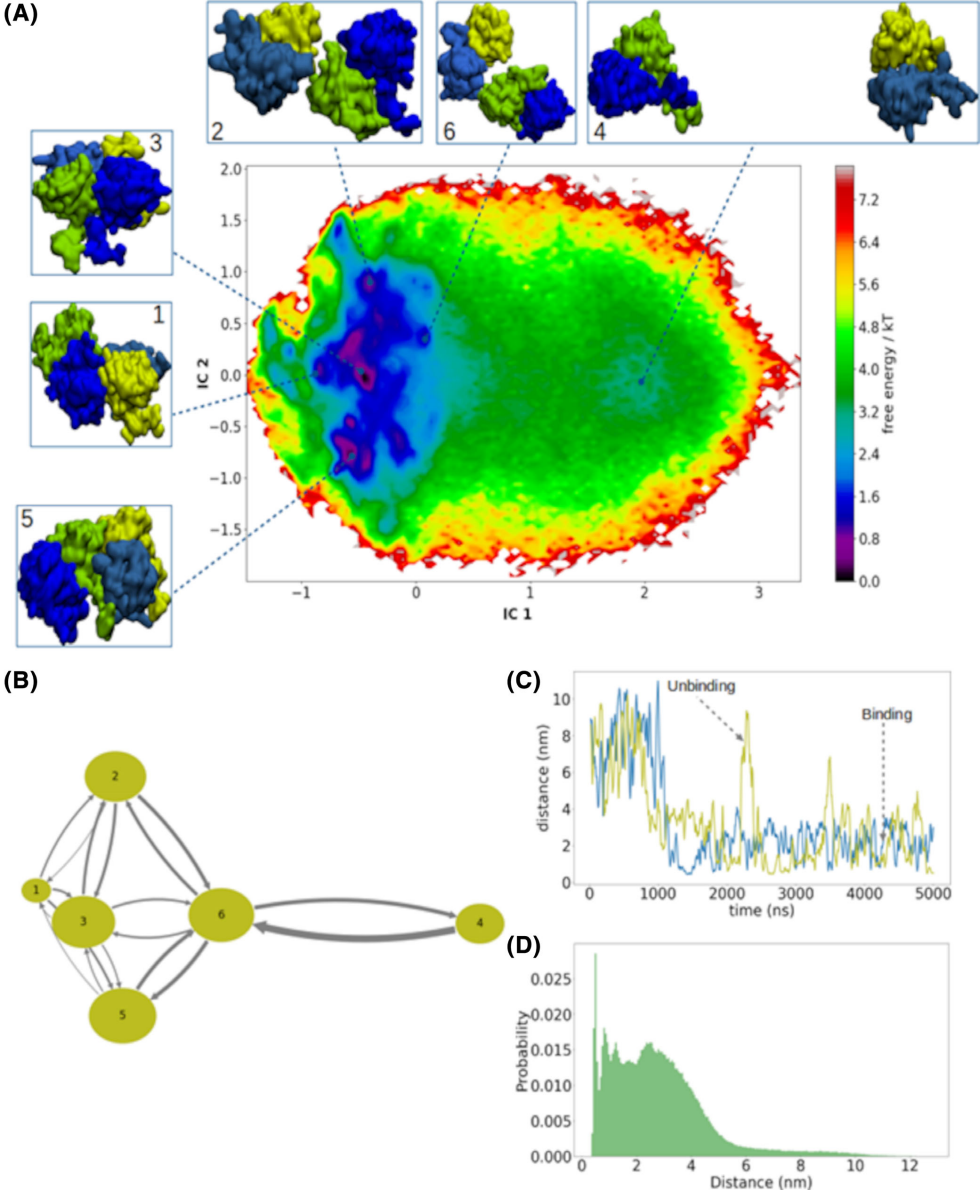
(A)Free energy surface and metastable states of the HβB2C (Q70E/Q162E), computed from the stationary MSM probability and projected onto the first two independent components (IC); also shown the representative structure of the relevant macrostates found in the of Q70E/Q162E dimerization process; The structures were colored, as follow: in green and yellow‐green represents the N‐terminal domain of each monomer, in blue and sky‐blue represents the C‐terminal domain of each monomer respectively. (B) Transition rates from each macrostate; the size of the circles represents the number of conformations in the respective macrostate, and the width of the arrows indicates the transitions between states, with their thickness proportional to the transition probability. (C) Two representative replicas indicating binding and unbinding events. (D) Fraction of conformations of all replicas at different distances.

Post‐translational modifications were associated with cataractogenesis.^46^, ^47^ Deamidation was observed in crystallins and related to aging cataracts.^48^ Experimental reports indicate that glutamine deamidation in HβB2C decreases protein stability.^49^ Previous work also reports that deamidation modify the electrostatic profile of the protein surface without complete crystallin unfolding.^21^ To investigate the influence of post‐translational modifications in HβB2C dimmer formation, we performed extensive coarse‐grained molecular dynamics (CG‐MD) of the HβB2C (Q70E/Q162E). As in wt‐HβB2C, the starting configuration of Q70E/Q162E was placed in the dissociated confirmation when each monomer separated ~8.0 nm. The number of replicas and the analysis were the same as performed for wt‐HβB2C. The free energy surface depicts six relevant macrostates for Q70E/Q162E (Figure 3A), five in dimeric and one macrostate in monomeric conformations (Figure 3A,B). We identify the macrostates 1, 3, and 5 as late dimers, macrostate 6 as an early dimeric stage. The macrostate 2 also can be reached from macrostate 6, however is not a late dimer yet. Macrostate 4 is in monomeric conformation. Interestingly, the early dimeric stage exhibits an interaction between the N‐terminal of one monomer and the C‐terminal of another (Figure 3A). The macrostate 2 shows an interaction between the N‐terminal of one monomer and the cleft of the hydrophobic interface of another monomer. The late dimers macrostates exhibit diverse conformations. The macrostates 1 and 3 show intercalated interactions between terminal domains of each monomer (i.e., the N‐terminal of one monomer is interacting with the C‐terminal of another monomer and vice versa) (Figure S9A,B). In macrostate 5, the N‐terminal domain of both monomers are interacting.

An evaluation of the association and dissociation events of Q70E/Q162E dimerization shows important changes compared with wt‐HβB2C profiles. Interestingly, the estimation of MFPT in the direction of macrostate 4 to 6 yield 57.6 ns, and in the direction of macrostate 6 to 4 is 1404.2 ns. Indicating that the formation of the early dimeric stage is ~24 folds faster than their dissociation (Figure 3B). This estimation suggests that the modification of the electrostatic surface of HβB2C, due to double deamidation, may help to favor the formation of more stable dimers. The alteration of the association and dissociation rates tends to the equilibrium to dimmer conformations. This observation may explain the first stages of crystallin aggregation due to post‐translational modifications. The binding and unbinding profiles can be observed by computation of the distance between each monomer as depicted in Figure 3C. We observed that the binding events are stable in most of the simulation time (~4 μs). However, the unbinding is a rare event and stable for only a few nanoseconds (Figure 3C). A high population in dimeric conformations (distance <6 nm) and a poorly populated in the monomeric conformations (distance >7 nm) were observed (Figure 3D).

A back‐conversion from CG to all‐atom was also performed on all macrostates of Q70E/Q162E. To evaluate the stability of late dimers, the macrostates 1, 3, and 5 were selected for all‐atom MD simulations. The computation of root‐mean square deviation (RMSD) shows that the dimers are stable over 50 ns of MD simulations (Figure S6B). Following this, the protein–protein contact maps were calculated. Interestingly, the contact map interactions are different compared with the wt‐HβB2C profile. The results show that macrostate 1 is mainly stabilized by at least eight interactions, including PRO38‐GLN184 and GLY35‐GLU138 (Figure S9A). The macrostate 3 include the ALA8‐ASP125, ILE124‐GLU77, and ASP125‐HIS195 interactions (Figure S9B). The macrostate 5 include the GLY28‐ASP127, GLU32‐HIS201, and GLU32‐PRO202 interactions (Figure S9C).

Our observations suggest that Q70E/Q162E late dimers have more interactions compared with wt‐HβB2C, with a contact fraction greater than 0.75. Interestingly, we do not observe interactions with GLU70 and GLU162 (Figure S9). In previous work, was described that this double deamidation change the electrostatic surface of HβB2C (Figure S7) and a slightly open structure was observed.^21^ This conformation exposes more residues on the protein surface.

Previous computational studies with γD‐crystallin, show that the N‐terminal domain is unfolded and is prone to aggregation through domain‐swapped conformations.^50^ Experiments in a redox condition showed that W42R and W42Q mutants of γD‐crystallin are prone to aggregation via domain‐swapped, and Monte Carlo simulations confirmed the presence of disulfide bonds with unfolded domains.^51^ Single‐molecule atomic force microscopy (AFM) experiments identified that the N‐terminal β‐hairpin is involved in domain swapping of γD‐crystallin.^52^ Coarse‐grained molecular dynamics of γD‐crystallin also identified domain‐swapped intermediates.^53^ However, SAXS experiments of the βB2‐crystallin demonstrated that face‐en‐face dimers and not swapped‐domain are the conformations observed in the solution environment.^16^ This finding was also observed in all‐atom and coarse‐grain molecular dynamics.^17^ Extensive all‐atom molecular dynamics simulations described that HβB2C (Q70E/Q162E) does not unfold, and only slight conformational changes were observed.^21^ Our results show that a complete unfold is not required to change the protein–protein association kinetics in the double deamidated HβB2C.

## 4 CONCLUSIONS

Human Crystallins are important to maintain the correct lens transparency. However, mutations and post‐translational modifications can modify their tertiary structure and promote the protein aggregation that manifests in cataract disease. Experimentally it has been observed that β‐crystallins can form oligomers. However, the molecular details of protein–protein associations remain elusive. This work employs extensive coarse‐grained molecular dynamics and a MSM to identify the first key stages of the HβB2C dimerization. We compared our results with the double deamidated HβB2C (Q70E/Q162E) system. The dimerization kinetics are different between these two systems. The deamidated system favors the dimeric states, suggesting that slightly modifying the protein surface can shift the equilibrium between monomeric and dimeric states. Suggesting the first stages of protein aggregation.

## AUTHOR CONTRIBUTIONS

**José‐Luis Velasco‐Bolom:** Investigation; writing – original draft; formal analysis; validation; methodology. **Laura Domínguez:** Conceptualization; investigation; methodology; writing – review and editing; resources; supervision; formal analysis.

## CONFLICT OF INTEREST STATEMENT

The authors declare no competing financial interest.

## PEER REVIEW

The peer review history for this article is available at https://www.webofscience.com/api/gateway/wos/peer-review/10.1002/prot.26547.

## Supporting information


**FIGURE S1:** All‐atom representation of macrostate 2 (wt‐HβB2C). In green colors we depict the N‐terminal of each monomer. In blue the C‐terminal of each monomer.
**FIGURE S2.** All‐atom representation of macrostate 1 (wt‐HβB2C). In green colors we depict the N‐terminal of each monomer. In blue the C‐terminal of each monomer.
**FIGURE S3.** All‐atom representations of macrostates 3, 4, and 5 (wt‐HβB2C); (A) macrostate 3, (B) macrostate 4, and (C) macrostate 5. In green colors we depict the N‐terminal of each monomer. In blue the C‐terminal of each monomer.
**FIGURE S4.** Chapman–Kolmogorov test of the wt‐HβB2C macrostates, comparing the probabilities between metastable states.
**FIGURE S5.** Chapman–Kolmogorov test of the HβB2C (Q70E/Q162E) macrostates, comparing the probabilities between metastable states.
**FIGURE S6.** RMSD of the three macrostates observed as late dimers. The RMSD values were computed over 50 ns of all‐atom molecular dynamic trajectory after backward conversion from CG. (A) wt‐HβB2C macrostates; (B) HβB2C (Q70E/Q162E) macrostates.
**FIGURE S7.** Electrostatic surface potential of the (A) wt‐HβB2C and (B) HβB2C (Q70E/Q162E).
**FIGURE S8.** Time‐averaged protein–protein contact maps of the wt‐HβB2C macrostates; (A) macrostate 1, (B) macrostate 2, and (C) microstate 4. The contact maps calculations were obtained from the all‐atom molecular dynamics of the late dimers. The contacts shown in the tables (right) were selected with a contact fraction greater than 0.75. A cutoff of 0.45 nm was used for contact maps.
**FIGURE S9.** Time‐averaged protein–protein contact maps of the HβB2C (Q70E/Q162E) macrostates; (A) macrostate 1, (B) macrostate 3, and (C) microstate 5. The contact maps calculations were obtained from the all‐atom molecular dynamics of the late dimers. The contacts shown in the tables (right) were selected with a contact fraction greater than 0.75. A cutoff of 0.45 nm was used for contact maps.

## Data Availability

The data that support the findings of this study are available from the corresponding author upon reasonable request.
