# Supplementary material for: Protein–protein association properties of human βB2‐crystallins
Source: Proteins. 2023 Jul 17;93(8):1361–8. doi: 10.1002/prot.26547 (PMC12260336; doi:10.1002/prot.26547)
Supplement: Supplementary file 1 — FIGURE S1: All‐atom representation of macrostate 2 (wt‐HβB2C). In green colors we depict the N‐terminal of each monomer. In blue the C‐terminal of each monomer. FIGURE S2. All‐atom representation of macrostate 1 (wt‐HβB2C). In green colors we depict the N‐terminal of each monomer. In blue the C‐terminal of each monomer. FIGURE S3. All‐atom representations of macrostates 3, 4, and 5 (wt‐HβB2C); (A) macrostate 3, (B) macrostate 4, and (C) macrostate 5. In green colors we depict the N‐terminal of each monomer. In blue the C‐terminal of each monomer. FIGURE S4. Chapman–Kolmogorov test of the wt‐HβB2C macrostates, comparing the probabilities between metastable states. FIGURE S5. Chapman–Kolmogorov test of the HβB2C (Q70E/Q162E) macrostates, comparing the probabilities between metastable states. FIGURE S6. RMSD of the three macrostates observed as late dimers. The RMSD values were computed over 50 ns of all‐atom molecular dynamic trajectory after backward conversion from CG. (A) wt‐HβB2C macrostates; (B) HβB2C (Q70E/Q162E) macrostates. FIGURE S7. Electrostatic surface potential of the (A) wt‐HβB2C and (B) HβB2C (Q70E/Q162E). FIGURE S8. Time‐averaged protein–protein contact maps of the wt‐HβB2C macrostates; (A) macrostate 1, (B) macrostate 2, and (C) microstate 4. The contact maps calculations were obtained from the all‐atom molecular dynamics of the late dimers. The contacts shown in the tables (right) were selected with a contact fraction greater than 0.75. A cutoff of 0.45 nm was used for contact maps. FIGURE S9. Time‐averaged protein–protein contact maps of the HβB2C (Q70E/Q162E) macrostates; (A) macrostate 1, (B) macrostate 3, and (C) microstate 5. The contact maps calculations were obtained from the all‐atom molecular dynamics of the late dimers. The contacts shown in the tables (right) were selected with a contact fraction greater than 0.75. A cutoff of 0.45 nm was used for contact maps. [file PROT-93-1361-s001.docx]

**Supporting Information**

Protein-protein association properties of human βB2-crystallins

José-Luis Velasco-Bolom^a^ and Laura Domínguez*^,a^

^a^ Facultad de Química, Departamento de Fisicoquímica, Universidad Nacional Autónoma de México, Mexico City 04510, Mexico

***Corresponding Author:** E-mail: [lauradd@unam.mx](mailto:lauradd@unam.mx)

*Table of contents*

| **Contents** |  | **Page** |
| --- | --- | --- |
| Fig. S1 | All-atom representation of macrostate 2 (wt-HβB2C) | S2 |
| Fig. S2 | All-atom representation of macrostate 1 | S3 |
| Fig. S3 | All-atom representations of macrostates 3, 4, and 5 | S4 |
| Fig. S4 | Chapman-Kolmogorov test of the wt-HβB2C | S5 |
| Fig. S5 | Chapman-Kolmogorov test of the HβB2C (Q70E/Q162E) | S6 |
| Fig. S6 | RMSD of the three macrostates observed as late dimers | S7 |
| Fig. S7 | Electrostatic surface potential | S7 |
| Fig. S8 | Time-averaged Protein-Protein contact maps of the wt-HβB2C | S8 |
| Fig. S9 | Time-averaged Protein-Protein contact maps of the  HβB2C (Q70E/Q162E) | S9 |


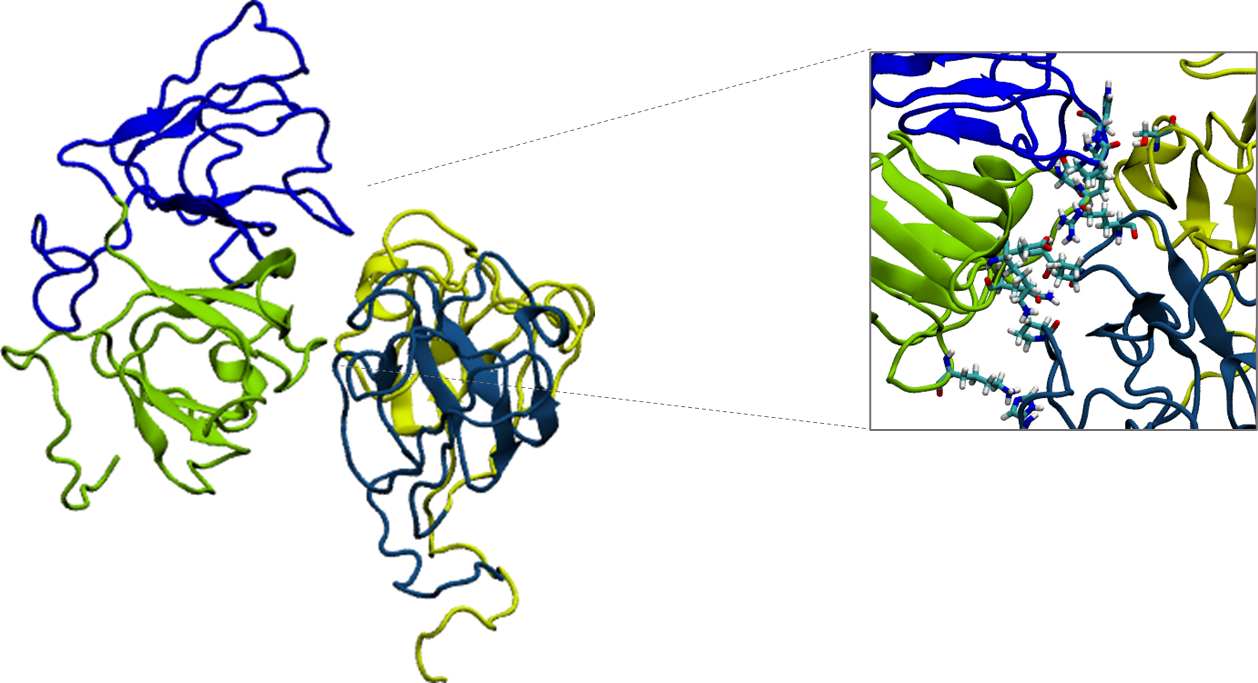


**Fig. S1** All-atom representation of macrostate 2 (wt-HβB2C). In green colors we depict the N-terminal of each monomer. In blue the C-terminal of each monomer.

**
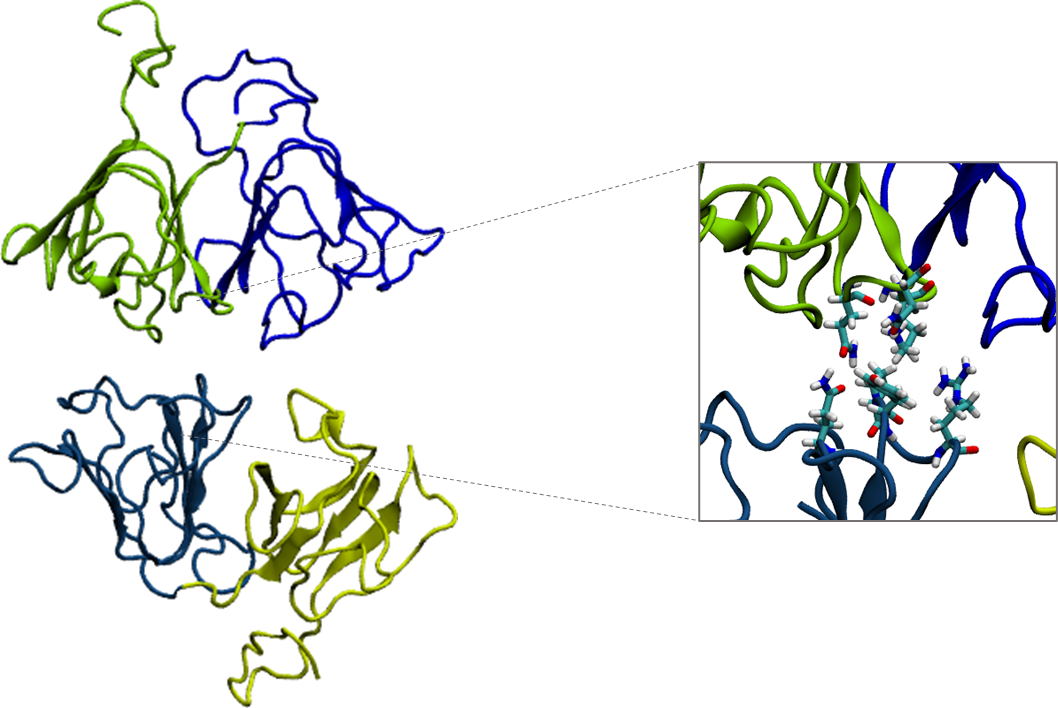
**

**Fig. S2** All-atom representation of macrostate 1 (wt-HβB2C). In green colors we depict the N-terminal of each monomer. In blue the C-terminal of each monomer.


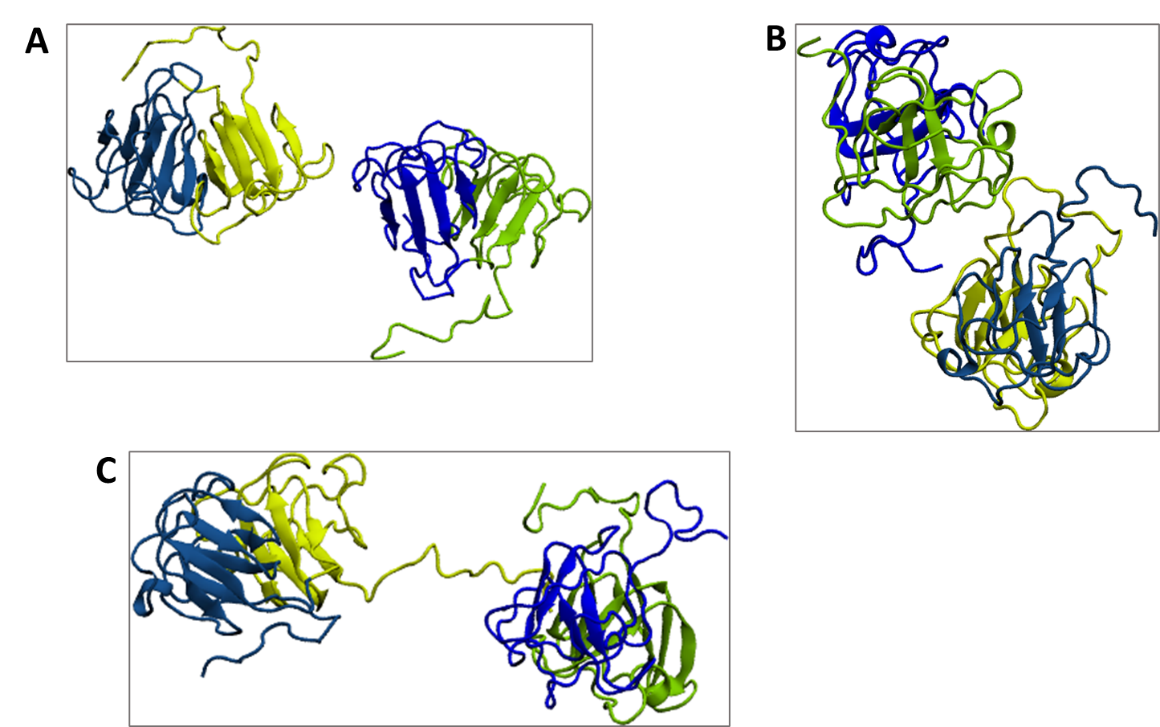


**Fig. S3** All-atom representations of macrostates 3, 4, and 5 (wt-HβB2C); A) macrostate 3, B) macrostate 4, and C) macrostate 5. In green colors we depict the N-terminal of each monomer. In blue the C-terminal of each monomer.


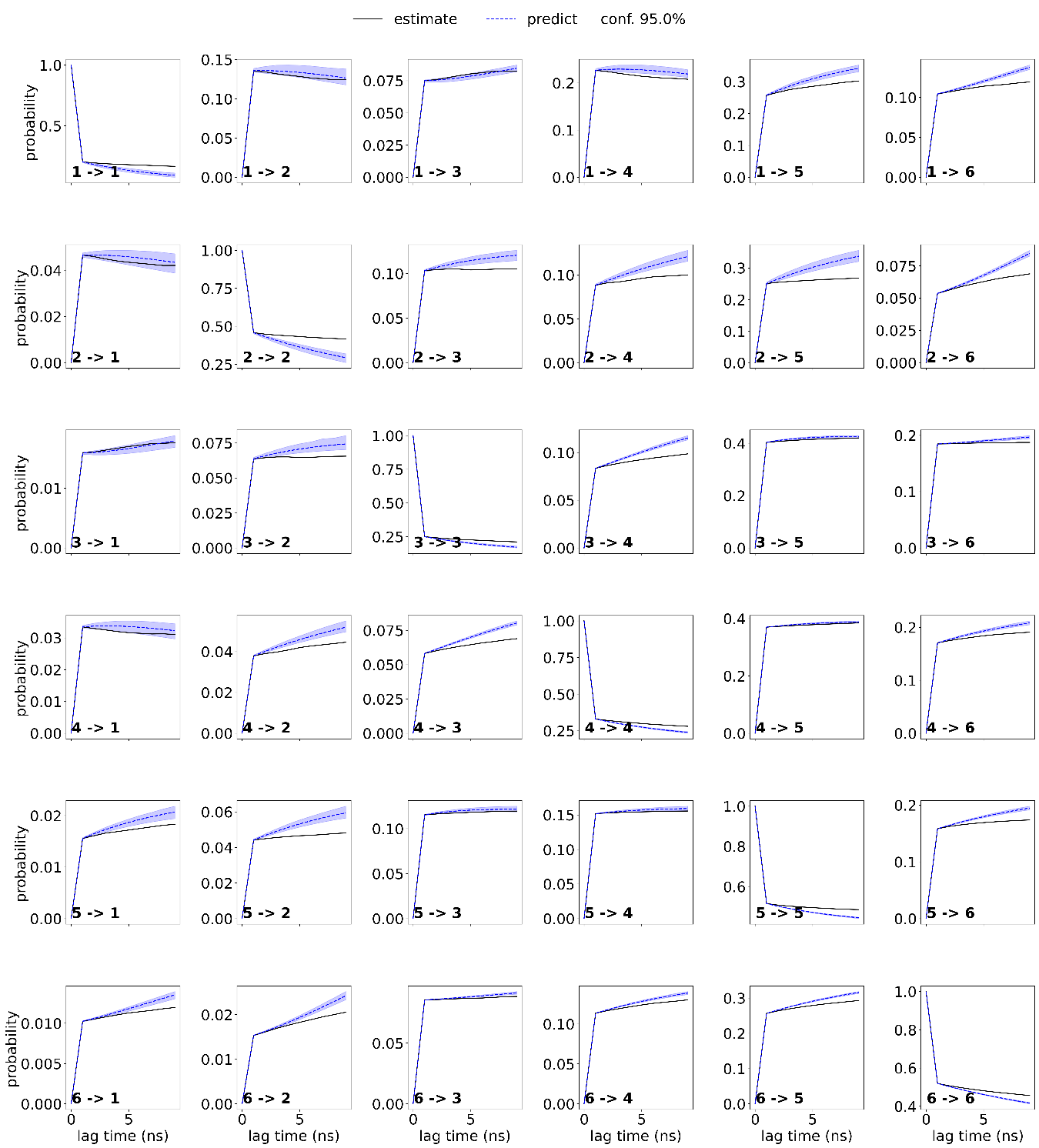


**Fig. S4** Chapman-Kolmogorov test of the wt-HβB2C macrostates, comparing the probabilities between metastable states.


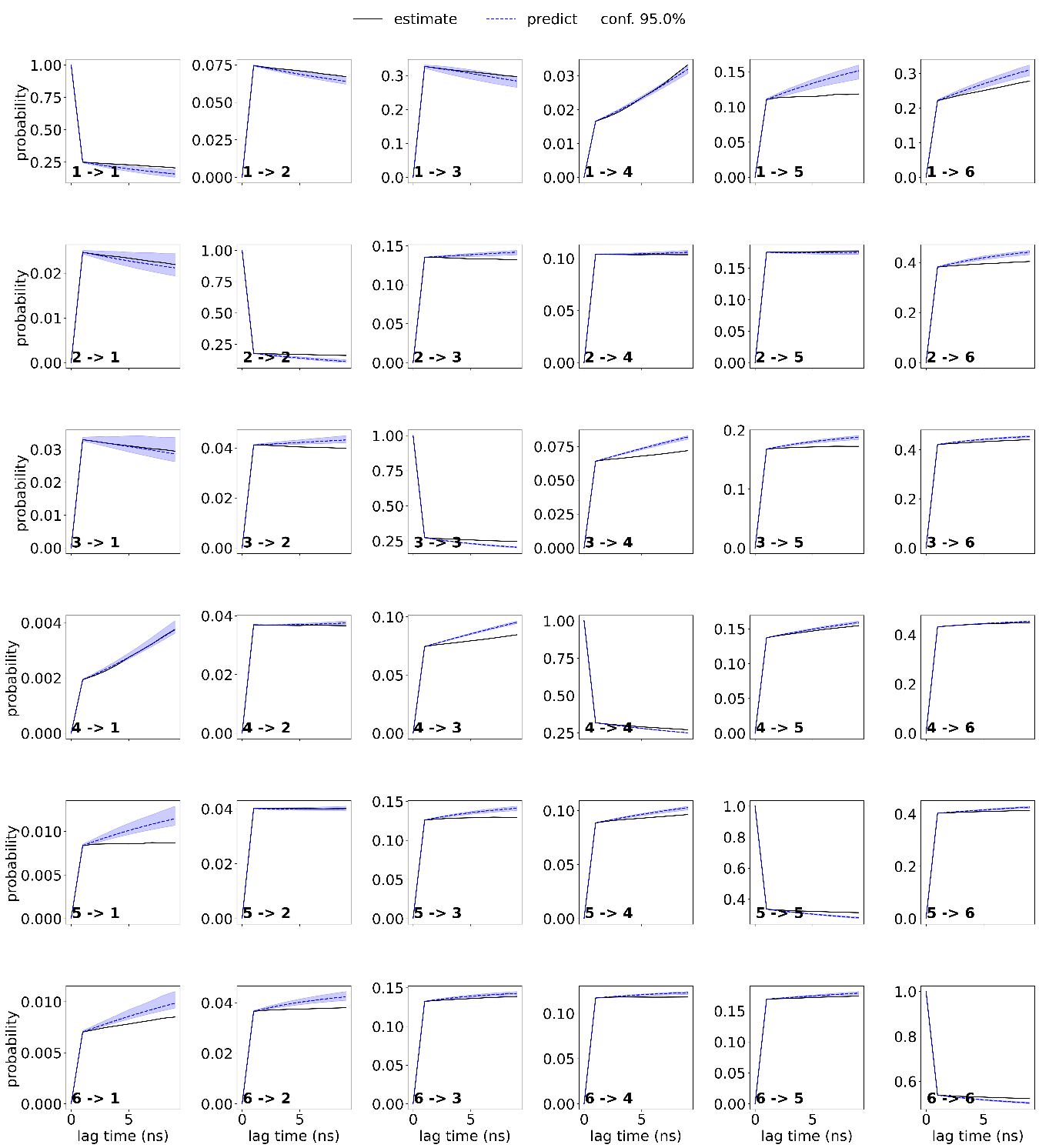


**Fig. S5** Chapman-Kolmogorov test of the HβB2C (Q70E/Q162E) macrostates, comparing the probabilities between metastable states.


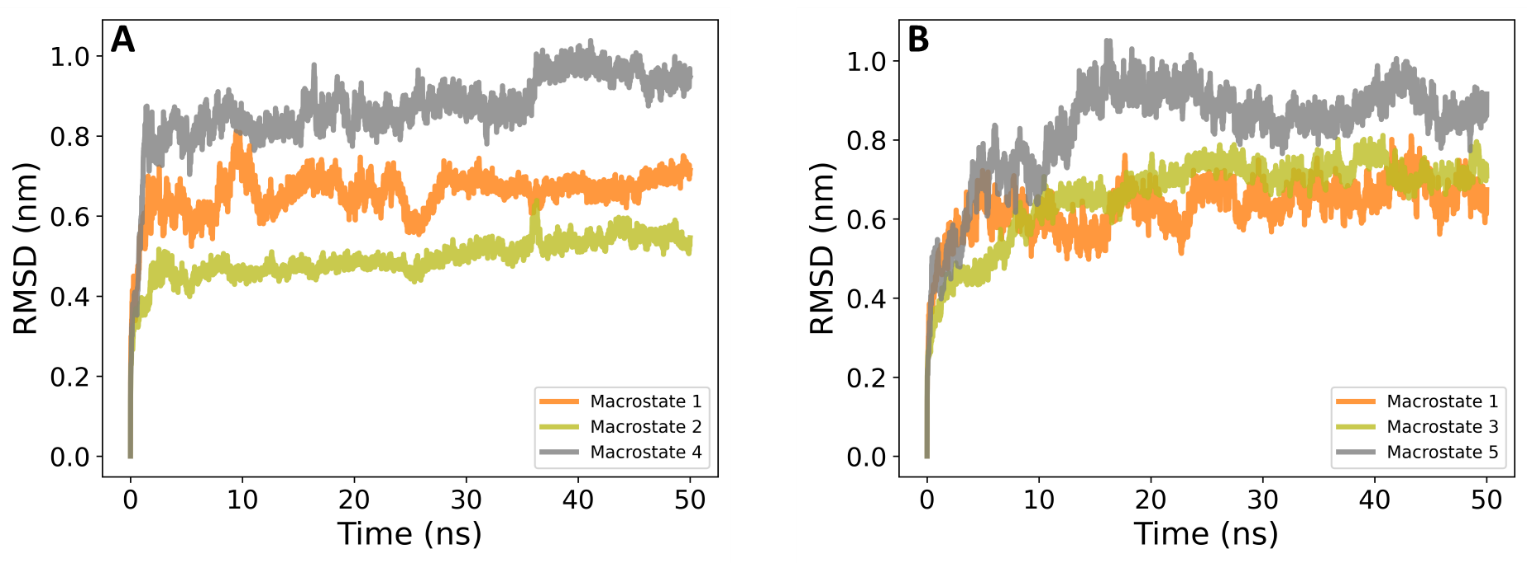


**Fig. S6** RMSD of the three macrostates observed as late dimers. The RMSD values were computed over 50 ns of all-atom molecular dynamic trajectory after backward conversion from CG. (A) wt-HβB2C macrostates; (B) HβB2C (Q70E/Q162E) macrostates.


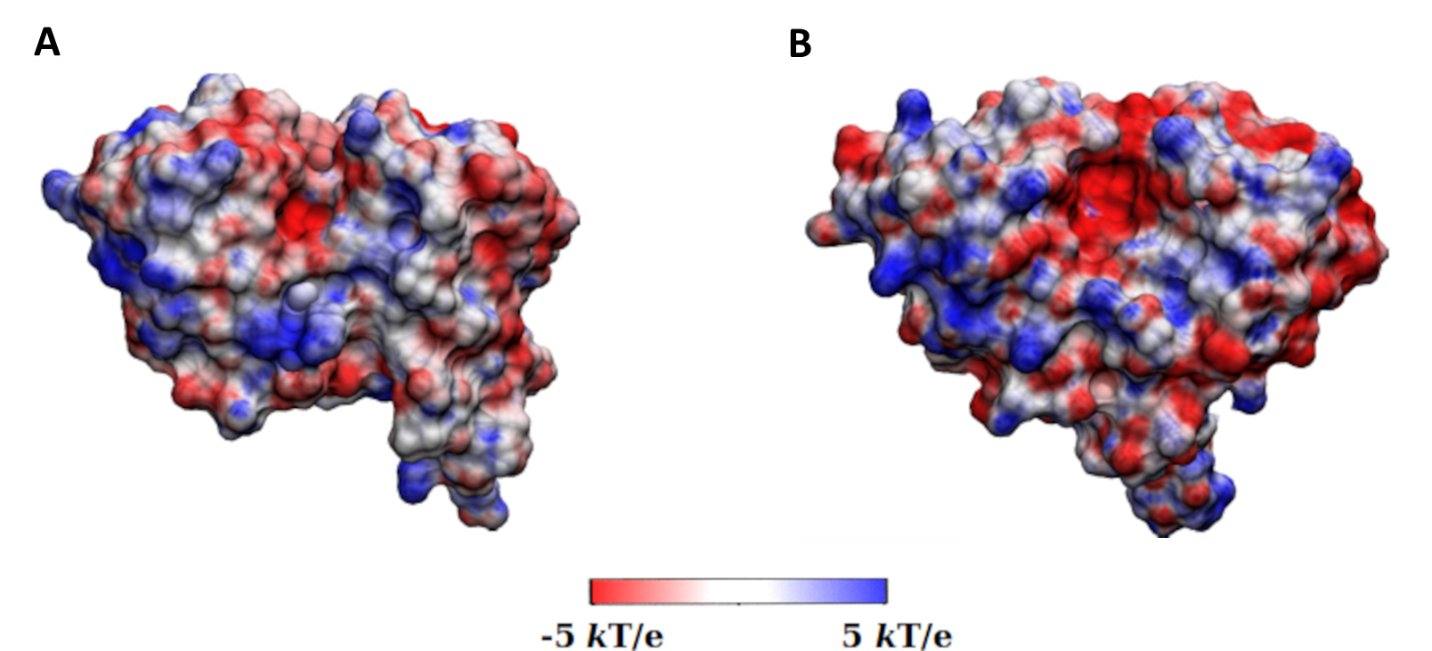


**Fig. S7** Electrostatic surface potential of the (A) wt-HβB2C and (B) HβB2C (Q70E/Q162E).

**
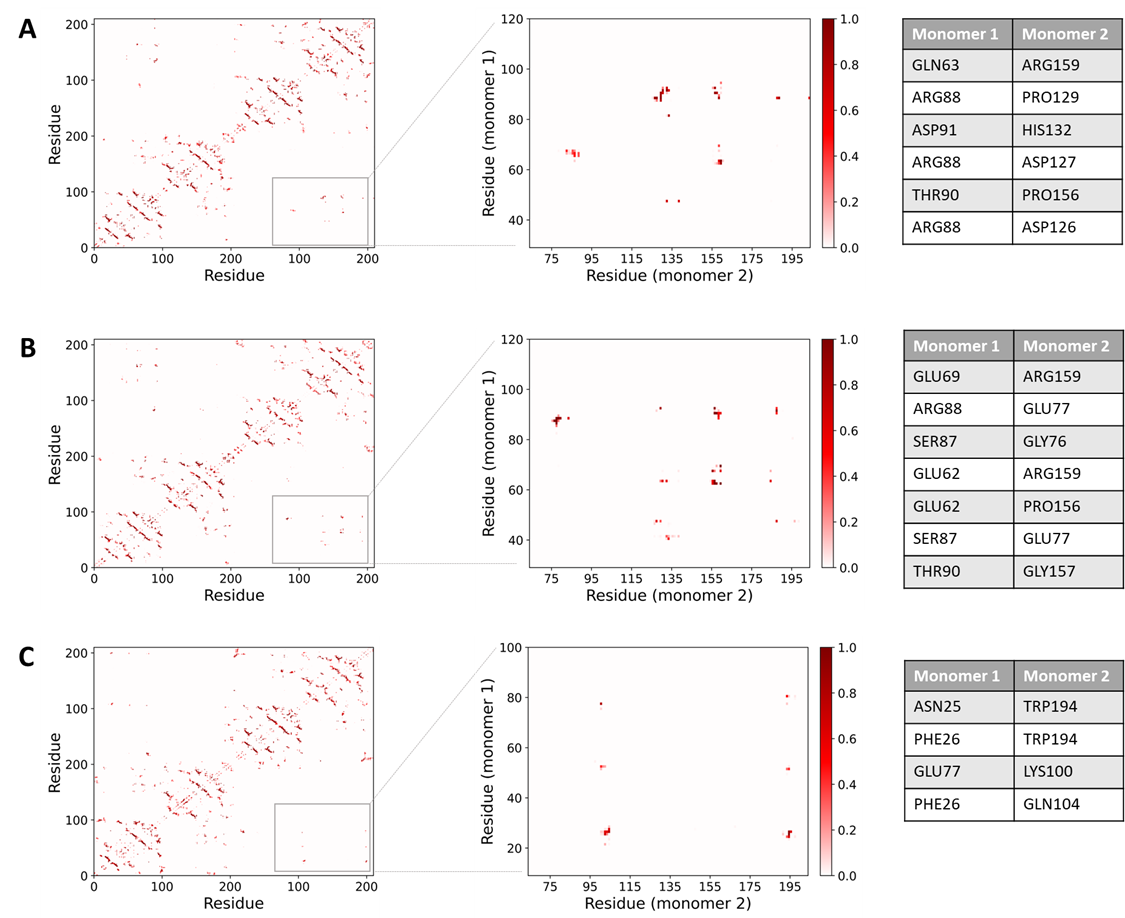
**

**Fig. S8** Time-averaged Protein-Protein contact maps of the wt-HβB2C macrostates; A) macrostate 1, B) macrostate 2, and C) microstate 4. The contact maps calculations were obtained from the all-atom molecular dynamics of the late dimers. The contacts shown in the tables (right) were selected with a contact fraction greater than 0.75. A cutoff of 0.45 nm was used for contact maps.


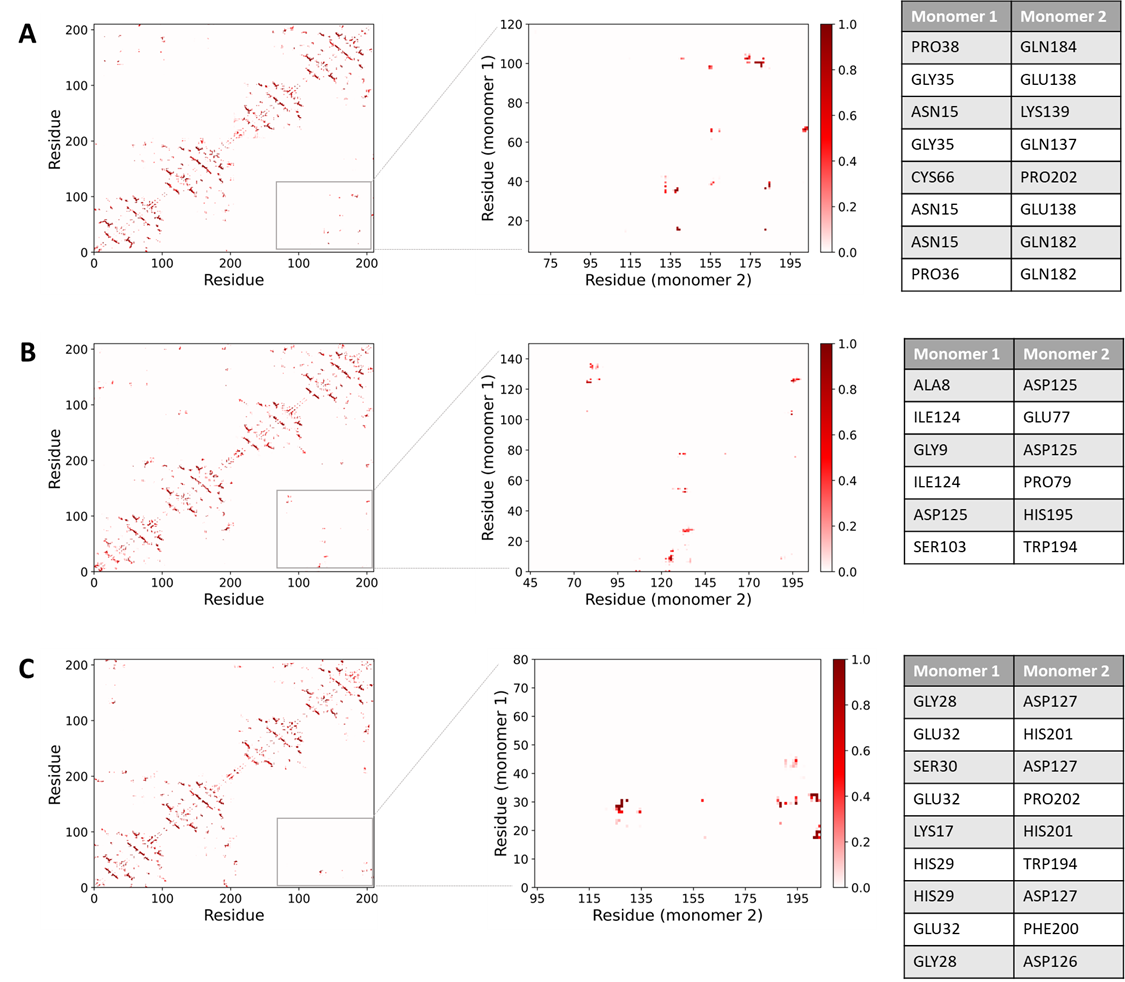


**Fig. S9** Time-averaged Protein-Protein contact maps of the HβB2C (Q70E/Q162E) macrostates; A) macrostate 1, B) macrostate 3, and C) microstate 5. The contact maps calculations were obtained from the all-atom molecular dynamics of the late dimers. The contacts shown in the tables (right) were selected with a contact fraction greater than 0.75. A cutoff of 0.45 nm was used for contact maps.
